# Zinc Downregulates HIF-1α and Inhibits Its Activity in Tumor Cells *In Vitro* and *In Vivo*

**DOI:** 10.1371/journal.pone.0015048

**Published:** 2010-12-13

**Authors:** Lavinia Nardinocchi, Valentina Pantisano, Rosa Puca, Manuela Porru, Aurora Aiello, Annalisa Grasselli, Carlo Leonetti, Michal Safran, Gideon Rechavi, David Givol, Antonella Farsetti, Gabriella D'Orazi

**Affiliations:** 1 Molecular Oncogenesis Laboratory, Department of Experimental Oncology, National Cancer Institute “Regina Elena”, Rome, Italy; 2 Chair of Endocrinology, Catholic University, Rome, Italy; 3 Department of Oncology and Experimental Medicine, School of Medicine, University “G. d'Annunzio”, Chieti, Italy; 4 Experimental Chemotherapy, National Cancer Institute “Regina Elena”, Rome, Italy; 5 National Research Council, Institute of Neurobiology and Molecular Medicine, Rome, Italy; 6 Cancer Research Center, Chaim Sheba Medical Center, Tel-Hashomer and Sachler School of Medicine, Tel-Aviv University, Tel-Aviv, Israel; 7 Department of Molecular Cell Biology, Weizmann Institute of Science, Rehovot, Israel; Institute for Medical Biomathematics, Israel

## Abstract

**Background:**

Hypoxia inducible factor-1α (HIF-1α) is responsible for the majority of HIF-1-induced gene expression changes under hypoxia and for the “angiogenic switch” during tumor progression. HIF-1α is often upregulated in tumors leading to more aggressive tumor growth and chemoresistance, therefore representing an important target for antitumor intervention. We previously reported that zinc downregulated HIF-1α levels. Here, we evaluated the molecular mechanisms of zinc-induced HIF-1α downregulation and whether zinc affected HIF-1α also *in viv*o.

**Methodology/Principal Findings:**

Here we report that zinc downregulated HIF-1α protein levels in human prostate cancer and glioblastoma cells under hypoxia, whether induced or constitutive. Investigations into the molecular mechanisms showed that zinc induced HIF-1α proteasomal degradation that was prevented by treatment with proteasomal inhibitor MG132. HIF-1α downregulation induced by zinc was ineffective in human RCC4 VHL-null renal carcinoma cell line; likewise, the HIF-1αP402/P564A mutant was resistant to zinc treatment. Similarly to HIF-1α, zinc downregulated also hypoxia-induced HIF-2α whereas the HIF-1β subunit remained unchanged. Zinc inhibited HIF-1α recruitment onto VEGF promoter and the zinc-induced suppression of HIF-1-dependent activation of VEGF correlated with reduction of glioblastoma and prostate cancer cell invasiveness *in vitro*. Finally, zinc administration downregulated HIF-1α levels *in vivo*, by bioluminescence imaging, and suppressed intratumoral VEGF expression.

**Conclusions/Significance:**

These findings, by demonstrating that zinc induces HIF-1α proteasomal degradation, indicate that zinc could be useful as an inhibitor of HIF-1α in human tumors to repress important pathways involved in tumor progression, such as those induced by VEGF, MDR1, and Bcl2 target genes, and hopefully potentiate the anticancer therapies.

## Introduction

The development of intratumoral hypoxia and angiogenesis is a hallmark of rapidly growing solid tumors [1]. The presence of hypoxia renders tumor cells resistant to conventional chemo- and radiotherapy selecting a more malignant and invasive phenotype and plays a negative role in patient prognosis [2]. Changes in gene expression in response to decreased oxygen availability are largely regulated by hypoxia inducible factor-1 (HIF-1), a heterodimeric transcription factor which consists of the HIF-1β subunit, constitutively expressed in cells, and the HIFs-α subunit whose stability is stimulated by low intracellular oxygen or genetic alteration [3]. There are two transactivating isoforms, HIF-1α and HIF-2α, whose expression and activity are tightly regulated by oxygen and appear to have complementary functions [3], [4]. In normoxia, HIF-1α is hydroxylated by prolyl hydroxylases (PHDs) at key proline residues (402 and 564) in the oxygen-dependent degradation domain (ODD) facilitating interaction to von Hippel-Lindau protein (pVHL), which in turn allows ubiquitination and subsequent HIF-1α degradation [5], [6]. Under hypoxic conditions, prolyl hydroxylation is inhibited, thereby stabilizing HIF-1α, which can then translocate into the nucleus and bind to constitutively expressed HIF-1β, forming the active HIF-1 complex [7]. Transition metals such as cobaltous ions can inhibit hydroxylation of HIF-1α and therefore induce elevated HIF-1α levels, mimicking hypoxia [3]. After dimerization with HIF-1β, HIF-1α binds to a promoter consensus sequence called hypoxia-responsive element (HRE), and controls the expression of several target genes involved in many aspects of cancer progression, including angiogenesis (e.g., vascular endothelial growth factor - VEGF), metabolic adaptation (e.g., Glut1), chemoresistance (e.g., MDR1), apoptosis resistance (e.g., Bcl2), invasion and metastasis (e.g., c-Met) [8]. Overexpression of HIF-1α has been found in many human cancers, including colon, brain, breast, gastric, lung, skin, ovarian, prostate, renal, and pancreatic carcinoma, and is associated with poor prognosis and failure of tumor treatment [8]. Likewise, overexpression of HIF-2α has been associated with bad prognosis in several tumors including prostate cancer [9] and also to regulate glioblastoma cancer stem cells in the hypoxic niche [10] that contributes to glioma radioresistance and tumor repopulation [11]. In tumor cells, HIF-1α can also be regulated by other genetic factors, such as oncogenes (Ras and phosphoinositide 3-kinase) or loss of tumor suppressors (VHL or PTEN) even under aerobic conditions. Inhibition of HIF-1α may, therefore, represent an attractive strategy with potential for synergism with other therapies [12], [13].

We recently reported a novel way to downregulate HIF-1α through transcriptional repression by HIPK2, a potential biomarker for tumor growth [14]. We found that HIPK2 binds and represses the HIF-1α promoter leading to inhibition of HIF-1 transcription of target genes, including VEGF, MDR1 and Bcl2 [15], [16]. Conversely, it has been shown that HIPK2 may be downregulated under hypoxia [17], interrupting the control feedback loop between HIPK2 and HIF-1α. Interestingly, we found that zinc supplementation to cancer cells restores the hypoxia-induced HIPK2 inhibition, leading to repression of HIF-1 pathway [18]. In this study we further analysed our observations and evaluated whether zinc could affect HIF-1α at protein level and which molecular mechanisms were involved. We used an *ex vivo* experimental model consisting of cell populations derived from explants of prostate cancer patients [19] characterized by a constitutively “hypoxic” phenotype (e.g., stabilized HIF-1α and HIF-2α protein in normoxia) associated with bad prognosis and a phenotype negative for HIF-1α and HIF-2α expression under aerobic condition associated with good prognosis [9], and a glioblastoma multiforme cell line which is a highly vascular tumor where expression of VEGF is hypoxia-induced by HIF-1-dependent transcriptional activation [20] and is relevant for glioblastoma aggressiveness and invasion [21], [22]. We show here that zinc may interfere with HIF-1α stabilization in human prostate cancer and glioblastoma cells following hypoxia, either induced or constitutive, leading to inhibition of HIF1-induced target genes, and reduction of both tube formation in HUVEC *in vitro* and tumor cell invasiveness, by chemoinvasion assay. Downregulation of HIF-1α in response to zinc was demonstrable *in vivo* in a xenograft human glioblastoma model in nude mice using noninvasive bioluminescent imaging. Therefore, zinc can be considered an interesting adjuvant in cancer therapy to target HIF-1α with the potential for disrupting multiple pathways crucial for tumor growth. This strategy could also improve the efficacy of established tumor therapies in solid tumors.

## Results

### Effect of zinc on HIF-1-induced VEGF expression and tube formation

**Figure 1:**
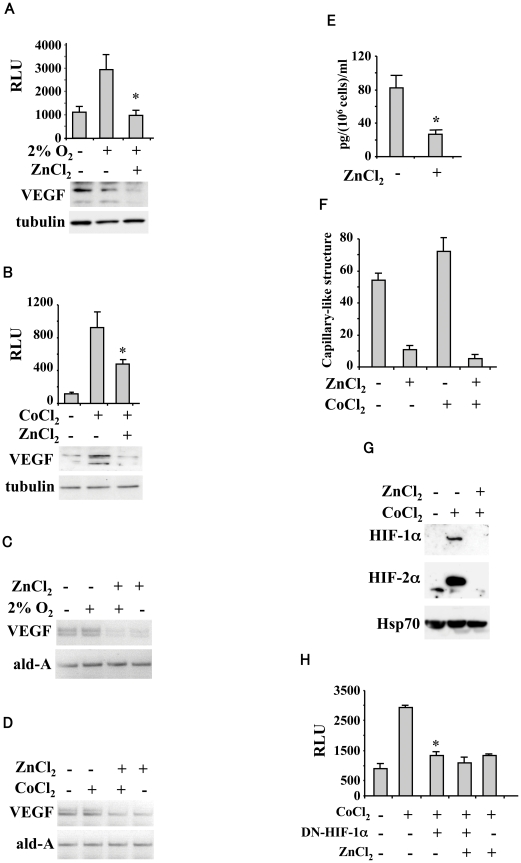
Effect of zinc on HIF-1-induced VEGF in glioblastoma and tube formation. (**A, upper panel**) U373MG glioblastoma cells were transfected with VEGF-luc reporter and 16 h thereafter treated with 100 µM ZnCl_2_ and 2% O_2_ for, respectively 24 and 16 h, before luciferase activity was assayed. Data are the mean ±S.D. of three independent experiments performed in duplicate. RLU: relative luciferase unit. *, P<0.005. (**A, lower panel**) Total cell extracts of cells treated as above were assayed for Western immunoblotting. Anti-tubulin was used as protein loading control. (**B**) U373MG glioblastoma cells were transfected with VEGF-luc reporter and 16 h thereafter treated with 100 µM ZnCl_2_ and 200 µM CoCl_2_ for, respectively 24 and 16 h, before luciferase activity was assayed. Data are the mean ±S.D. of three independent experiments performed in duplicate. RLU: relative luciferase unit. *, P<0.005. (**B, lower panel**) Total cell extracts of cells treated as in (A) for Western immunoblotting. (**C**) Total mRNAs were reverse transcribed from U373MG cells treated as in (A), for semi-quantitative RT-PCR analysis of VEGF expression. Aldolase (ald-A) was used as internal control. (**D**) Total mRNAs were reverse transcribed from U373MG cells treated as in (B), for semi-quantitative RT-PCR analysis of VEGF expression. Aldolase (ald-A) was used as internal control. (**E**) Serum-starved U373MG cells were cultured for 24 h with 100 µM ZnCl_2_, and cell conditioned media were analyzed by ELISA assay for VEGF secretion. ELISA results (mean ±S.D.) for duplicates from four independent experiments are shown. *, P<0.005 compared with the untreated control. (**F**) HUVECs were incubated at 37°C on Matrigel with CM form U373MG cells untreated or treated with ZnCl_2_ (100 µM) in normoxia or under hypoxia (CoCl_2_ 200 µM for 24 h). The number of tube networks from triplicate wells (10 fields/well) was quantified at ×20 magnification after 3 hours of differentiation. (**G**) U373MG cells were treated with 100 µM ZnCl_2_ and 200 µM CoCl_2_ for, respectively 24 and 16 h and nuclear cell extracts were assayed for Western immunoblotting. Anti-Hsp70 was used as protein loading control. (**H**) U373MG cells were transfected with HIF-1α dominant negative vector (DN-HIF-1α, 2 µg) and 16 h after transfection treated with 100 µM ZnCl_2_ and 200 µM CoCl_2_ for, respectively 24 and 16 h. Luciferase activity was assayed 36 h thereafter and normalized by β-galactosidase activity. Data represent mean ±S.D. of three independent experiments performed in duplicate. *, P<0.001.

Our first experiments tested the effect of zinc on HIF-1-responsive VEGF-luc activity in human U373MG glioblastoma cells treated with hypoxia (2% O_2_ or cobalt chloride to mimic hypoxia). The results of luciferase assay show that the hypoxia-induced (Figure 1A) as well as the cobalt-induced VEGF-luc (Figure 1B) activity was strongly inhibited by zinc. Parallel inhibitory effect was observed with the VEGF mRNA levels (Figure 1C, D) as well as with the hypoxia- and cobalt-induced VEGF protein levels (Figure 1A, 1B lower panels) and VEGF secretion by ELISA assay (Figure 1E). To examine whether the effect of zinc on VEGF expression was associated with endothelial cell morphogenesis, the growth of human umbilical vein endothelial cells (HUVEC) was evaluated *in vitro* on Matrigel in the presence of cell-conditioned media (CM) of U373MG cells untreated or treated with zinc in normoxia and after cobalt chloride treatment. As shown in Figure 1F, the CM from U373MG cells provoked a positive effect on the tube formation of HUVEC which increased further following cobalt treatment. The tube formation was strongly abolished by zinc supplementation either in normoxia and hypoxia (Figure 1F). As cobalt stabilized HIF-1α and to a larger extent also HIF-2α levels and they were both repressed by zinc (Figure 1G), we evaluated the requirement of HIF-1α for VEGF inhibition by transducing U373MG cells with an expression vector encoding the dominant negative form of HIF-1α without DNA binding and transactivation domains (HIF-1αDN) [23]. Inhibition of HIF-1α by HIF-1αDN vector strongly impaired the cobalt-induced VEGF-luc activity that was not further reduced by zinc (Figure 1H). Altogether, these data show that zinc inhibited VEGF expression and tube formation induced by glioblastoma CM and that HIF-1α was required for zinc-induced VEGF downregulation.

### Zinc downregulates HIF-1α and inhibits HIF-1 transcriptional activity

**Figure 2:**
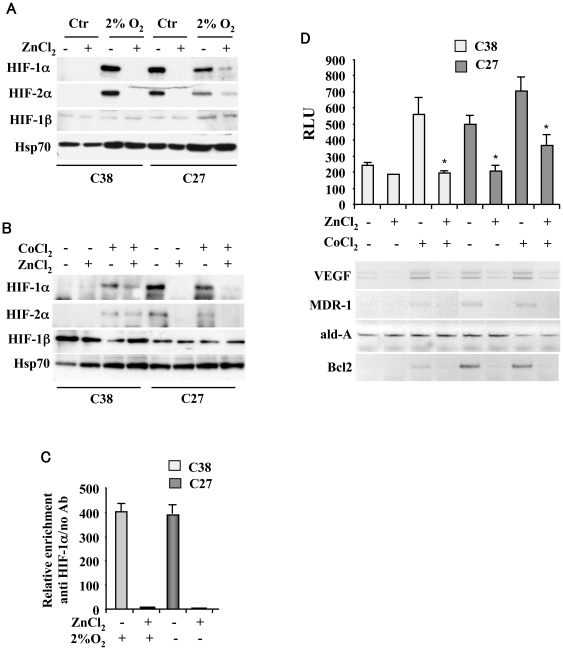
HIFs-α destabilization and inhibition of HIF-1 transcriptional activity by zinc. (**A**) Proliferating C38 and C27 prostate cancer cells were treated with 100 µM ZnCl_2_ and 2% O_2_ for, respectively 24 and 16 h. Equal amount of nuclear cell extracts were assayed for Western immunoblotting. Anti-Hsp70 was used as protein loading control. (**B**) Proliferating C38 and C27 prostate cancer cells were treated with 100 µM ZnCl_2_ and 200 µM CoCl_2_ for, respectively 24 and 16 h. Equal amount of nuclear cell extracts were assayed for Western immunoblotting. Anti-Hsp70 was used as protein loading control. (**C**) ChIP analysis with anti-HIF-1α antibody or no antibody as control was performed in C38 cells treated with 100 µM ZnCl_2_ and 2% O_2_ for, respectively 24 and 16 h and in C27 cells treated with100 µM ZnCl_2_ for 24 h under basal “hypoxic” condition. Recruitment of HIF-1α onto the VEGF promoter was detected by qRT-PCR, using primers spanning the HRE region. Relative enrichment of HIF-1α compared to no antibody onto VEGF promoter is shown. The data represent the mean of 2 independent experiments ±S.D. (**D, upper panel**) C38 and C27 cells were transfected with VEGF-luc reporter and 16 h after transfection treated with 100 µM ZnCl_2_ and 200 µM CoCl_2_ for, respectively 24 and 16 h, before luciferase activity was assayed. Data represent the mean ±S.D. of three independent experiments performed in duplicate. RLU: relative luciferase unit. *, P<0.005. (**D, lower panel**) Total mRNAs were reverse transcribed from C38 and C27 cells treated as above for semi-quantitative RT-PCR analyses of HIF-1 target genes. Aldolase (ald-A) is shown as internal control.

To elucidate the molecular mechanism by which zinc affects the HIF-1/VEGF signalling, we took advantage of an *ex vivo* experimental model consisting of cell populations derived from explants of prostate cancer patients [19] characterized by a “constitutively hypoxic” phenotype (e.g., stabilized HIF-1α and HIF-2α protein in normoxia, namely C27) associated with bad prognosis and a phenotype negative for HIF-1α and HIF-2α expression under aerobic condition associated with good prognosis (namely C38) [9]. In C38 cells, HIF-1α is undetectable under normoxia as previously reported [9] and the hypoxia (2% O_2_)-stabilized HIF-1α levels were strongly decreased by zinc (Figure 2A). In C27 cells, the HIF-1α levels, stabilized in basal “constitutively hypoxic” condition as previously reported [9], were strongly suppressed following 16 h zinc treatment both in basal condition and under hypoxia (Figure 2A). Zinc abolished also the levels of HIF-2α which like HIF-1α dimerizes with HIF-1β and activates hypoxia-induced transcription, while had no effect on HIF-1β levels (Figure 2A). Similar results were obtained with cobalt chloride treatment which inhibits hydroxylation, mimicking hypoxia (Figure 2B). As a result of HIF-1α downregulation, zinc abolished the hypoxia-induced HIF-1α recruitment onto its binding sites in the VEGF promoter in C38 cells and in basal “constitutively hypoxic” condition in C27 cells (Figure 2C); similarly, zinc inhibited the hypoxia-induced HIF-1α recruitment onto hTERT promoter (not shown) which has been previously shown to be regulated by HIFs-α in the prostate cancer model [9]. The zinc-inhibited HIF-1 transcriptional activity was next analysed by using a transient reporter assay in which endogenous HIF-1 binds hypoxia response elements (HREs) of VEGF gene promoter cloned upstream of a luciferase transcriptional reporter [24]. The hypoxia-driven VEGF-luc activity in both C38 and C27 cells as well as the VEGF-luc activity in C27 cells under basal “constitutively hypoxic” condition were significantly inhibited by zinc (Figure 2D) which, on the other hand, did not affect the activity of a VEGF-luc vector mutated in the HIF-1 binding site (not shown). Consequently, the levels of mRNAs of HIF-1 target genes such as VEGF, MDR-1 and Bcl2 were significantly diminished by zinc in both cell lines (Figure 2C, lower panel), in agreement with our previous results on human colon cancer cells [18]. These findings indicate that zinc downregulated both HIF-1α and HIF-2α and impaired HIF-1α recruitment onto target genes inhibiting HIF-1 transcriptional activity affecting multiple pathways crucial for tumor growth.

### Zinc induces HIF-1α proteasomal degradation

**Figure 3:**
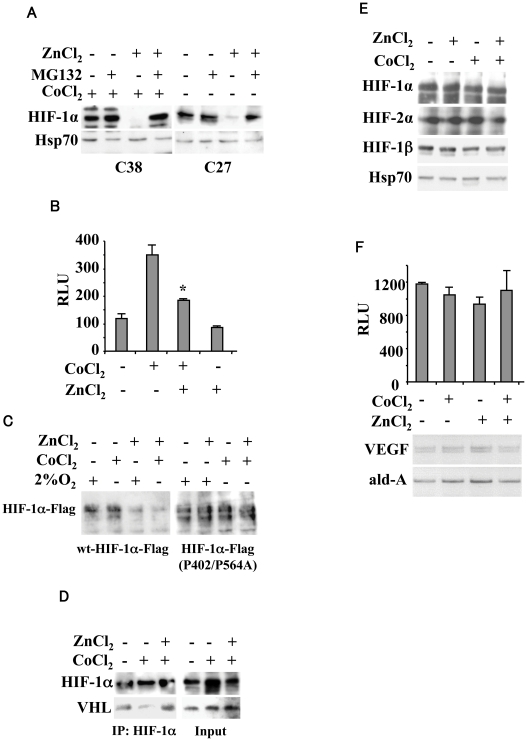
Zinc induces HIF-1α proteasomal degradation. (**A**) C38 and C27 cells were treated with 100 µM ZnCl_2_ for 24 h in the presence or absence of 10 µM proteasome inhibitor MG132 for 4 h, under hypoxia (200 µM CoCl_2_ for 16 h) for C38 cells and in basal “hypoxic” condition for C27 cells. Equal amount of nuclear cell extracts were assayed for Western immunoblotting. Anti-Hsp70 was used as a protein loading control. (**B**) 293 cells were transfected with HIF-1α-ODD-luc vector and 16 h after transfection treated with 100 µM ZnCl_2_ and 200 µM CoCl_2_ for, respectively 24 and 16 h, before luciferase activity was assayed. Data represent the mean ±S.D. of three independent experiments performed in duplicate. RLU: relative luciferase unit. *, P<0.005. (**C**) C38 cells were transfected with either HIF-1α-Flag expression vector or the HIF-1αP402/P564A-Flag mutant and 16 h after transfection treated with 100 µM ZnCl_2_ for 24 h and 2% O_2_ or 200 µM CoCl_2_ for 16 h. Equal amount of nuclear cell extracts were assayed for Western immunoblotting with anti-Flag antibody. (**D**) C38 cells were treated with 100 µM ZnCl_2_ and 200 µM CoCl_2_ for, respectively 24 and 16 h, in the presence of 10 µM proteasome inhibitor MG132 for 4 h. Equal amount of total cell extracts were immunoprecipitated with anti-HIF-1α antibody and then assayed for Western immunoblotting to detect pVHL interaction. Inputs represent 1/10 of total cell extracts used for immunoprecipitation. (**E**) RCC4 VHL-null renal carcinoma cells were treated with 100 µM ZnCl_2_ and 200 µM CoCl_2_ for, respectively 24 and 16 h. Equal amount of nuclear cell extracts were assayed for Western immunoblotting. Anti-Hsp70 was used as protein loading control. (**F**) RCC4 VHL-null renal carcinoma cells were transfected with VEGF-luc reporter and 16 h after transfection treated with 100 µM ZnCl_2_ and 200 µM CoCl_2_ for, respectively 24 and 16 h, before luciferase activity was assayed. Data represent the mean ±S.D. of three independent experiments performed in duplicate. RLU: relative luciferase unit. In the lower panel, total mRNAs were reverse transcribed from RCC4 cells for semi-quantitative RT-PCR analysis of VEGF. Aldolase (ald-A) was used as internal control.

HIF-1α levels are regulated by oxygen-dependent prolyl hydroxylases enzymes (PHDs) which hydroxylate prolyl residues 402 and 564 in its oxygen-dependent degradation (ODD) domain, marking HIF-1α for recognition by VHL and targeting it for proteasomal degradation [5], [6]. To determine whether zinc promotes HIF-1α proteasomal degradation, C38 and C27 cells were treated with proteasome inhibitor MG132. The zinc-downregulated HIF-1α levels, under hypoxia in C38 cells and in basal “constitutively hypoxic” condition in C27 cells, were completely rescued by treatment with protease inhibitor MG132 (Figure 3A). To further test our hypothesis, we used a plasmid encoding a fusion protein consisting of the oxygen-dependent degradation domain of HIF-1α (aa 530–652) fused to the N terminus of firefly luciferase (ODD-luc). This plasmid behaves like HIF-1α in living cells and can bind to pVHL oncosuppressor for HIF-1α degradation, and the intensity of the luciferase signal is directly proportional to inhibition of the prolyl hydroxylation in the HIF-1α-ODD domain [25]. As shown in Figure 3B, cobalt treatment induced the ODD-luciferase activity whereas zinc significantly decreased it, counteracting the effect of cobalt. Hence, to evaluate the role of prolyl hydroxylation in the sensitivity of HIF-1α to zinc, we tested a Flag-tagged HIF-1α expression vector with prolyl mutations P402A and P564A (HIF-1αP402/P564A) [26]. As shown in Figure 3C, zinc strongly abolished the hypoxia-induced (by using either 2% O_2_ or cobalt) wt-HIF-1α levels as expected; on the contrary, the HIF-1αP402/P564A mutant appeared to be resistant to zinc treatment. Then we tested the HIF-1α/VHL interaction since pVHL only binds to HIF-1α after the latter is enzymatically hydroxylated on prolyl residues within the ODD [5], [6]. As shown in Figure 3D, the HIF-1α/VHL protein-protein interaction was reduced by about 80% under hypoxia while it was strongly reconstituted by zinc. In agreement, endogenous HIF-1α protein levels were not downregulated by zinc in the RCC4 VHL-null renal carcinoma cells [27] either in aerobic condition nor following cobalt treatment (Figure 3E). Similarly, HIF-2α levels were not downregulated by zinc in the RCC4 cells (Figure 3E). Finally, the HIF-1-responsive VEGF-luc activity (Figure 3F, upper panel) as well as the VEGF mRNA levels (Figure 3F, lower panel), already elevated in RCC4-VHL-null cells under basal condition and not further induced by cobalt, were not downregulated by zinc. Altogether, these data support the hypothesis that zinc promoted HIF-1α proteasomal degradation likely through prolyl hydroxylation within the ODD and VHL.

### Zinc decreases tumor cell invasiveness *in vitro* and induces HIF-1α downregulation *in vivo*

**Figure 4:**
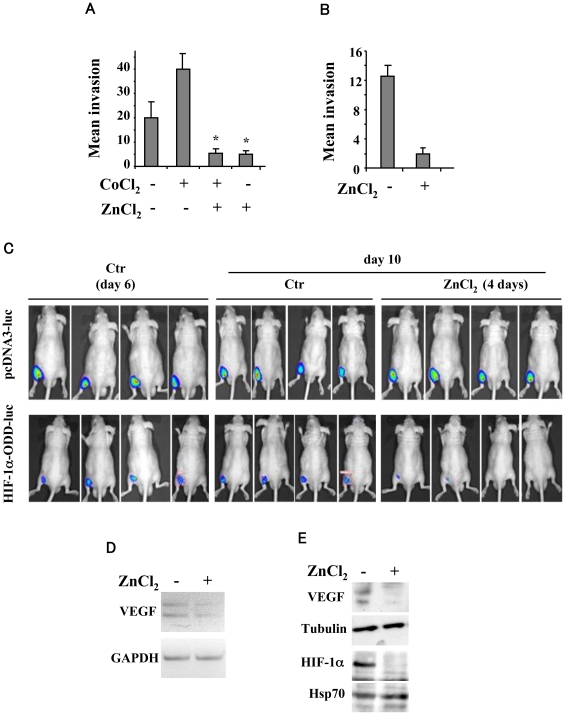
Zinc decreases tumor cell invasiveness *in vitro* and induces HIF-1α downregulation *in vivo*. (**A**) Serum-starved U373MG cells were treated with 100 µM ZnCl_2_ and 200 µM CoCl_2_ for, respectively 24 and 16 h and cell invasion was measured using a Boyden's chamber invasion assay. Cell invasion results (mean ±S.D.) for quadruplicates from four independent experiments are shown. *, P<0.0001. (**B**) Serum-starved C27 cells were treated with 100 µM for 24 h, under basal “hypoxic” condition and cell invasion was measured as in (A). Cell invasion results (mean ±S.D.) for quadruplicates from four independent experiments are shown. *, P<0.0001. (**C**) Representative tumors derived from human U373MG cells transfected with HIF-1α-ODD-luc and pcDNA3-luc control vectors marked with luciferase were imaged using the IVIS imaging system 200 series at day 6 after tumor cell injection and at day 10 following 4 days of zinc daily administration. Four mice/group are shown. (**D**) RNA samples from explanted tumors, at day 10 after tumor cell injection and after 4 days of zinc treatment, were used for reverse-transcription (RT)-PCR. The mRNA levels were normalized to GAPDH expression. (**E**) Tissue samples as in (D) were used for Western immunoblotting of VEGF and HIF-1α levels and anti-tubulin and anti-Hsp70 were used, respectively, as protein loading control. Similar results were obtained with different tissue samples.

Next, we tested the biological relevance of zinc by Boyden chamber invasion assay and by *in vivo* bioluminescence imaging. As shown in Figure 4A, the ability of U373MG cells to invade was strongly induced by cobalt and significantly inhibited by zinc. Interestingly, zinc significantly inhibited U373MG cell invasiveness also in basal condition, which correlated with the zinc-induced inhibition of VEGF mRNA and protein secretion (Figure 1C, D, E) and with the finding that impairment of VEGF pathway has been shown to reduce tumor cell invasiveness [21], [22]. Similar inhibition of cell invasiveness was also observed with the “constitutively hypoxic” C27 prostate cancer cells (Figure 4B).

Finally, to test whether zinc was able to affect HIF-1α also *in vivo*, we took advantage of the bioluminescence imaging technique. To this aim, human U373MG cells were transfected with the HIF-1α-ODD-luc or the pcDNA3-luc control vectors and implanted subdermally into the flanks of athymic nude mice. When the tumors reached approximately 200 mm^3^ (about one week after injection for both groups), mice were randomized and subsequently imaged after intraperitoneal administration of luciferin, before starting zinc treatment with daily administration. As shown in Figure 4C, four days of zinc treatment significantly suppressed HIF-1α-ODD-luc, levels, compared to the HIF-1α-ODD-luc untreated mice, as measured by bioluminescence (*P* = 0.0033 by Student *t* test). Conversely, zinc did not affect control pcDNA3-luc levels (*P* = 0.3240), indicating specific zinc action on HIF-1α. The reduction of HIF-1α-ODD-luc after zinc treatment correlated with strong reduction of intratumoral VEGF mRNA levels (Figure 4D) as well as with abrogation of VEGF and HIF-1α intratumoral protein levels (Figure 4E). Collectively, these observations indicate that zinc was able to inhibit tumor cell invasiveness *in vitro*. More importantly, zinc was able to downregulate HIF-1α levels also *in vivo*, impairing its transcriptional activity.

## Discussion

In this study we analysed the molecular mechanisms by which zinc downregulated HIF-1α levels and activity in prostate cancer and glioblastoma cells under hypoxia, whether constitutive or induced, and its biological relevance. We found that: *i*) zinc induced HIF-1α proteasomal degradation; *ii*) zinc downregulated HIF-1α *in vitro* and *in vivo* by using bioluminescence imaging; *iii*) zinc inhibited HIF-1-induced VEGF expression and tube formation; *iv*) zinc-induced VEGF inhibition correlated with suppression of cell invasiveness.

The observation that zinc induced HIF-1α protein downregulation was corroborated by the use of MG132 proteasome inhibitor that indeed counteracted the HIF-1α protein degradation (Figure 3A). The effect of zinc on HIF-1α proteasomal degradation is in agreement with previous observations showing that a zinc chelator (namely Clioquinol) can stabilize the HIF-1α protein in normoxia by blocking its ubiquitination, and that addition of Zinc(II) reverses its effects [28]. Moreover, it has been shown that antioxidants such as ascorbic acid, N-acetylcysteine and vitamin C decrease HIF-1α levels by acting on PHD, likely by maintaining the catalytic ferrous ion of PHD in the reduced active state, and by inducing VHL-mediated HIF-1α degradation [29]. Although we did not directly measure prolyl hydroxylation in our model, we showed that zinc was ineffective in downregulating HIF-1α in the presence of HIF-1α expression vector with prolyl mutations P402A and P564A (HIF-1αP402/P564A) (Figure 3C) and in the absence of VHL (in RCC4-VHL null cells) (Figure 3E), suggesting an involvement of prolyl hydroxylation and VHL.

Of note, we found that zinc inhibited HIF-2α other than HIF-1α. This is relevant in light of the fact that recent studies in neuroblastomas have shown that HIF-2α may be the main regulator of long term hypoxic gene expression and thus confer a more malignant phenotype [30]. Another study recently established the existence of a hypoxic niche that through HIF-2α regulates glioblastoma cancer stem cells [10] that contribute to glioma radioresistance and tumor repopulation [11], rendering this kind or tumor very aggressive and almost incurable. Moreover, both HIF-1α and HIF-2α cooperate in the activation of a prognostic transcriptional pattern delineating an aggressive tumor in the human prostate cancer model that we used in this study [9], rendering both molecules interesting targets for anticancer intervention and zinc a valid tool.

The effect of zinc on HIF-1α stability was further evaluate *in vivo* by using bioluminescence imaging. We found that a reporter consisting of the oxygen-dependent degradation domain of HIF-1α (aa 530–652) fused to the N terminus of firefly luciferase (ODD-luc) [25] was responsive to zinc treatment *in vitro* and *in vivo*. To the best of our knowledge, this is the first time that has been shown that intratumoral HIF-1α levels were almost completely abrogated following 4 days of zinc treatment, by bioluminescence imaging (Figure 4C). The specificity of zinc action of HIF-1α was confirmed by Western immunoblotting of tumor tissue extracts (Figure 4E). In tumor xenografts, decreased HIF-1 activity is usually associated with a slower growing and less angiogenic tumor phenotype [31]–[33], as also assessed by HIF-1α inhibition in a model of inducible RNA interference *in vivo* [34]. However, we have previously shown that zinc treatment alone does not greatly affect tumor growth *per se*, at least with the dose and administration scheduling that we used, rather it changes the activity of transcription factors (e.g., p53 and HIPK2) important to inhibit tumor growth and restore/improve chemosensitivity [18], [35]. Here, we give a mechanistic explanation of zinc function on HIF-1α protein regulation and rationalize the use of zinc in combination with conventional antitumor therapies since zinc inhibited HIF-1α-induced transcription of target genes important for chemoresistance such as MDR1 and Bcl2 (Figure 2D).

Finally we showed that zinc could inhibit the HIF-1-induced VEGF expression which correlated with reduced tumor cell invasiveness and reduced tube formation (Figure 1 and Figure 4). This is of interest in light of the fact that multiple angiogenesis inhibitor have been therapeutically validated in preclinical cancer models and clinical trials [36]. However, angiogenesis inhibitors targeting the VEGF pathway have recently demonstrated antitumor effect in glioblastoma model but concomitantly select for resistant cells that yield tumor invasiveness and metastasis, due to activation of HIF-1 pathway [37], [38]. Therefore, our results may be pertinent to translational considerations by targeting both VEGF and HIF-1 in solid tumors with hypoxic regions or in highly angiogenic tumors such as glioblastomas. Thus, abrogation of both HIF-1α and VEGF protein levels was evidenced in tissue tumor extracts by Western immunioblotting (Figure 4E). Further studies in orthotopic models for instance of brain tumors are needed to exploit the potential application of zinc derivatives as adjuvant with classical antitumoral treatments to prevent the effects of hypoxia-induced HIF-1 pathway, restore sensitivity to drugs and suppress tumors.

## Materials and Methods

### Ethics Statement

All animals were handled in strict accordance with good animal practice as defined by the relevant national and/or local animal welfare bodies, and in accordance with the Italian and European legislation. All work was performed in accordance with the guidelines of the National Cancer Institute “Regina Elena”, where there is currently no active Ethical Committee for animal research, and has been filed with the Veterinary Service Unit and the Italian Ministry of Health, in accordance with the Italian and European legislation.

### Cell lines and treatments

Human prostate C38 and C27 cancer cells [9], [19], human embryo kidney 293, RCC4 VHL-null renal cancer cells [27] were maintained in DMEM (Life Technology-Invitrogen), while human glioblastoma U373MG cell line (ATCC) was maintained in RPMI-1640 (Life Technology-Invitrogen), all supplemented with 10% heat-inactivated fetal bovine serum plus glutamine and antibiotics. Cell treatments were as follow: 100 µM zinc chloride (ZnCl_2_), 200 µM hypoxia mimetic cobalt chloride (CoCl_2_), and 2% O_2_ for the indicated times. Proteasome inhibitor MG132 (Biomol, Research Laboratories) was added to the culture medium to a final concentration of 10 µmol/L for 4 h.

### Western immunoblotting and co-immunoprecipitation

Total cell extracts and nuclear extracts were prepared essentially as described [18], [39]. For HIF-1α/VHL co-immunoprecipitation C38 cells were treated with 100 µM ZnCl_2_ and 200 µM CoCl_2_ for, respectively 24 and 16 h, in the presence of 10 µM proteasome inhibitor MG132 for 4 h. Total cell extracts were prepared by incubating at 4°C for 30 min in lysis buffer (20 mmol/L Hepes, 100 mmol/L NaCl, 5 mM EDTA (pH 8.0), 10% glicerol). Following preclearing for 1 h at 4°C, immunoprecipitation was performed by incubating total cell extracts with anti-HIF-1α antibody pre-adsorbed to protein G-Agarose (Pierce), rocking for 2 h at 4°C. The beads were then resuspended in 5× Laemmli buffer and subjected to Western blot with the indicated primary antibodies. Proteins were transferred to a polyvinylidene difluoride (PVDF) membrane (Millipore). Immunoblottings were performed with the following antibodies: mouse monoclonal anti-HIF-1α, anti-HIF-2α and anti-HIF-1β, (Novus Biologicals), rabbit polyclonal anti-VEGF (A-20) (Santa Cruz Biotechnology), rabbit polyclonal anti-VHL (Cell Signaling), mouse monoclonal anti-tubulin (Immunological Sciences), and mouse monoclonal anti-Hsp70 (Stressgene). Immunoreactivity was detected by enhanced chemiluminescence kit (ECL; Amersham).

### Transfection, plasmids and transactivation assay

Luciferase activity was measured in C27, C38 and 293 cells transiently transfected using respectively the cationic polymer LipofectaminePlus method (Invitrogen) according to manufacturers' instructions and the N,N-bis-(2- hydroxyethyl)-2-amino-ethanesulphonic acid-buffered saline (BBS) version of the calcium phosphate procedure [40]. The plasmid reporter used were the VEGF-luc vector that contains the hypoxia response elements (HREs) of VEGF gene promoter cloned upstream of a luciferase transcriptional reporter and the VEGF-luc reporter mutated in the HRE sites [24] (kindly provided by C. Gaetano, IDI, IRCCS, Rome, Italy), the ODD-luc plasmid that encodes fusion proteins consisting of HIF-1α (aa 530–652, a unique oxygen-dependent degradation domain (ODD) fused to the N terminus of firefly luciferase [25]; the expression vectors encoding the dominant negative form of HIF-1α without DNA binding domain and transactivation domain (pCEP4-HIF-1αDN) [23] (kindly provided by B.H. Jiang, Nanjing Medical University, China), the Flag-tagged HIF-1α expression vector and the Flag-tagged HIF-1α with prolyl mutations P402A and P564A [26] (kindly provided by G.L. Semenza, The Johns Hopkins University School of Medicine, Baltimore, MD, USA) were also used. The amount of plasmid DNA in each sample was equalized by supplementing with empty vector. Transfection efficiency was normalized with the use of a co-transfected β-galactosidase (β-gal) plasmid. Luciferase activity was assayed on whole cell extract and the luciferase values were normalized to β-galactosidase activity and protein content and expressed as relative luciferase unit (RLU).

### RNA extraction and reverse transcription (RT)-PCR analysis

Cells and tumors were harvested in TRIzol Reagent (Invitrogen) and total RNA was isolated following the manufacturer's instructions essentially as described [35] by using genes specific oligonucleotides under conditions of linear amplification. PCR was performed in duplicate in two different sets of cDNA. PCR products were run on a 2% agarose gel and visualized by ethidium bromide staining using UV light. The housekeeping aldolase (ald-A) mRNA, used as internal control.

### Chromatin Immunoprecipitation (ChIP) assays and Real-time PCR

Chromatin immunoprecipitation assays (ChIP) and real-time PCR was performed as reported [41]. DNA fragments were recovered and analyzed by qRT-PCR. Standard curves were generated by serially diluting the input (5-log dilutions in triplicate). qRT-PCR was done in the ABI Prism 7500 and 7900HT fast PCR instruments (Applied Biosystems) using SYBR Master mix (Applied Biosystems) with evaluation of dissociation curves. The qRT-PCR analyses were performed in duplicate and the data obtained were normalized to the corresponding DNA input control. Data are represented as relative enrichment (with values for no antibody being subtracted from those with antibody).

### ELISA and chemoinvasion assay

The VEGF protein levels in the cell-conditioned medium were determined in triplicate, in 16 h serum-starved U373MG cells, by enzyme-linked immunosorbent assay (ELISA) using the Quantikine human VEGF immunoassay kit (R&D Systems, Minneapolis, MN) according to manufacturer's instructions. VEGF levels were normalized to cell number and expressed as pg VEGF/10^6^ cells/ml.

Chemoinvasion was assessed using a 48-well modified Boyden chamber (NeuroProbe, Pleasanton, CA) and 8-µm pore polyvinyl pyrrolidone-free polycarbonate Nucleopore filters (Costar, New York, NY), as reported [42]. The filters were coated with an even layer of 2 mg/ml Matrigel (BD Biosciences). The lower compartment of chamber was filled with chemoattractants (20% FBS) or with 0.1% BSA as negative control. Serum-starved cells (2×10^4^ cells) pre-treated with ZnCl_2_ in the presence or absence of cobalt were harvested and placed in the upper compartment (40 µl/well) of the Boyden chamber. After 24 h of incubation at 37°C the filters were removed and then stained with Diff-Quick (Biomap, Milan,Italy Merz-Dade, Dudingen, Switzerland), and the migrated cells in 40 high power fields were counted. Each experimental point was analyzed in quadruplicate.

### Morphogenesis assay on Matrigel

Conditioned media (CM) was prepared by incubating subconfluent U373MG cells in serum-free medium in the presence of ZnCl_2_ (100 µM) and CoCl_2_ (200 µM) for 24 h before harvesting. Endothelial cell differentiation by using the in vitro Matrigel assay was performed as previously described [43]. Briefly, a 24-microwell plate was filled with 100 µl/well of unpolimerized Matrigel (BD Biosciences, USA) and allowed to polymerize for 1 h at rool temperature under air flow. Human umbilical vein endothelial cells (HUVEC) (4×10^4^ cells/well) were plated into wells containing CM of U373 cells left untreated or treated with cobalt and zinc. The number of tubular structures from triplicate wells (10 fields/well) was quantified at ×20 magnification after 4 h of differentiation. Each experiment was performed in triplicate.

### *In vivo* HIF-1α-ODD-luc imaging

CD-1 male nude (nu/nu) mice, 6–8 weeks old and weighting 22–24 g were purchased from Charles River Laboratories (Calco, Italy). They were housed in specific pathogen-free conditions and fed standard cow pellets and water ad libitum. Studies were performed in accordance with institutional standard guidelines for animal experiments. Human U373MG glioblastoma cells were transfected with pcDNA3-luc and HIF-1α-ODD-luc vectors and 24 h after transfection, 5×10^6^ viable cells were injected i.m. on the flank of each mouse suspended in 0.1 mL PBS. The mice were examined every day after injection until solid tumors reached approximately 200 mm^3^ weight (6 days from injection). Mice were then randomized in two groups (4 mice/group) as follow: 1) PBS, as control; 2) ZnCl_2_ (10 mg zinc/kg body weight). ZnCl_2_ was administrated once daily by oral administration, over the course of 4 days.

Luciferase expression was quantified *in vivo* in relative units with a bioluminescence imaging system (IVIS imaging system 200, Caliper Life Sciences, Hopkiton, MA, USA). This imaging system was used to measure intratumoral hypoxia in each mouse tumor according to the degree of reaction between the luciferase enzyme (the reporter of ODD-luc activity) and a standardized luciferin substrate stock solution (Caliper Life Sciences). Repeated images of luciferase expression were acquired according to the manufacturer's specified protocol. Imaging was performed at baseline (day 6 after tumor cell injection) before daily administration of ZnCl_2_. The images were then acquired 4 days after zinc administration. During each imaging session, the mice were anesthetized with a combination of tiletamine-zolazepam (telazol, Virbac, Carros, France) and xylazine (Xilazyne/Rompun BAYER) given i.m. at 2 mg/Kg. Then mice were injected i.p. with 150 mg/kg D-luciferin (Caliper Life Sciences) and imaged in the supine position and 10–15 min after luciferin injection. Data were acquired and analyzed using Living Image software version 3.0 (Caliper Life Sciences).

### Statistical analyses

All experiment unless indicated were performed at least three times. All experimental results were expressed as the arithmetic mean and standard deviation (S.D.) of measurements was shown. Student's *t*-test was used for statistical significance of the differences between treatment groups. Statistical analysis was performed using analysis of variance at 5% (p<0.05) or 1% (p<0.01).
